# [Expression of Concern] Inhibition of TMEM45A suppresses proliferation, induces cell cycle arrest and reduces cell invasion in human ovarian cancer cells

**DOI:** 10.3892/or.2026.9185

**Published:** 2026-08-24

**Authors:** Jing Guo, Li Chen, Ning Luo, Weihong Yang, Xiaoyan Qu, Zhongping Cheng

Oncol Rep 33: 3124–3130, 2015; DOI: 10.3892/or.2015.3902

Following the publication of the above article, a concerned author drew to the authors' attention that the western blot data showing the TGF β1-experiments in Fig. 4C and D on p. 3129 for the HO-8910 and A2780 cell lines were strikingly similar, suggesting that the data in this figure had been assembled incorrectly. In addition, various of the western blots featured in this figure, and also in Fig. 2B on p. 3127, were remarkably similar to data which were subsequently submitted for publication in articles written by different authors at different research institutes to a number of different journals, at least one of which has since been retracted on account of data sharing issues with other papers.

The authors have been contacted by the Editorial Office to offer an explanation for the apparent anomalies in the presentation of the western blot data in this paper, and we are awaiting their response. Owing to the fact that the Editorial Office has been made aware of potential issues surrounding the scientific integrity of this paper, we are issuing an Expression of Concern to notify readers of this potential problem while the Editorial Office continues to investigate this matter further.

